# Growth differentiation factor 15 as a novel diagnostic and therapeutic marker for autoimmune hepatitis

**DOI:** 10.1038/s41598-022-12762-9

**Published:** 2022-05-24

**Authors:** Teruko Arinaga-Hino, Tatsuya Ide, Jun Akiba, Hiroyuki Suzuki, Reiichiro Kuwahara, Keisuke Amano, Toshihiro Kawaguchi, Tomoya Sano, Eisuke Inoue, Hironori Koga, Keiichi Mitsuyama, Yasutoshi Koga, Takuji Torimura

**Affiliations:** 1 Division of Gastroenterology, Department of Medicine, Kurume University School of Medicine, 67 Asahi-machi, Kurume, Fukuoka 830-0011 Japan; 2 Department of Diagnostic Pathology, Kurume University Hospital, Kurume, Fukuoka Japan; 3 Showa University Research Administration Center, Shinagawa-ku, Tokyo Japan; 4 Cognitive and Molecular Research Institute of Brain Diseases, Kurume University School of Medicine, Kurume, Fukuoka Japan

**Keywords:** Biomarkers, Gastroenterology

## Abstract

Growth differentiation factor 15 (GDF15) has been reported to be associated with fibrosis and cancer in liver disease. Diagnosis of autoimmune hepatitis (AIH) is often difficult because of the lack of specific markers. We investigated whether GDF15 is useful for diagnosing AIH and determined its therapeutic effects. We enrolled 171 Japanese patients as follows: AIH (n = 45), hepatitis B (HB) (n = 17), hepatitis C (HC) (n = 15), primary biliary cholangitis (PBC) (n = 20), and 74 healthy controls. Serum GDF15 levels were measured, and immunohistological analyses of GDF15 were performed using liver tissue of AIH patients. (1) GDF15 levels (pg/ml) were higher in AIH (1994.3 ± 1258.0) and HC (1568.0 ± 822.3) than in HB (953.2 ± 871.4), PBC (643.9 ± 247.0), and controls (475.3 ± 145.3) (*p* < 0.0001), as well as in cirrhosis patients (n = 31) than in non-cirrhosis patients (n = 66) (1926.6 ± 1026.0 vs. 1249.1 ± 1124.1, *p* < 0.0001). In non-cirrhosis patients, GDF15 levels were higher in AIH (1914.0 ± 1327.2) than in HC (955.7 ± 502.7), HB (519.3 ± 197.5), and PBC (643.9 ± 247.0) (*p* < 0.0001). (2) GDF15 was positively correlated with M2BPGi (r = 0.7728), total bilirubin (r = 0.6231), and PT-INR (r = 0.6332). (3) GDF15 levels could be used to distinguish AIH from other liver diseases in non-cirrhosis patients, with an area under the curve of 0.9373 (sensitivity 93.6%, specificity 79.3%, cut-off value 931.3). (4) GDF15 in AIH decreased after treatment. (5) Immunohistological analyses in AIH liver tissues revealed that GDF15 was strongly expressed in inflammatory cells, hepatic cytoplasm, and sinusoidal endothelial cells, but decreased after treatment. GDF15 is a novel diagnostic marker for AIH and is also expected to be a therapeutic marker for AIH.

Clinical Trials Registration: The study protocol was approved by the institutional review board of Kurume University (Approval No.: 19049).

## Introduction

Autoimmune hepatitis (AIH) is a chronic inflammatory liver disease of unknown etiology, and it progresses to cirrhosis and liver failure if diagnosis or treatment is delayed or ineffective. Therefore, prompt diagnosis and early treatment are important. AIH is characterized by elevated transaminases, increased immunoglobulin G levels, histological features of interface hepatitis with lymphocyte and plasma cell infiltration, and presence of autoantibodies in serum^1^. However, because AIH has no disease-specific diagnostic markers, it is diagnosed using the International AIH Group (IAIHG) scoring system^2,3^, and diagnostic criteria are based on disease characteristics^4^ or exclusion of other liver diseases. Notably, the prevalence of atypical AIH, such as acute onset cases^5,6^, drug-induced cases^7,8^, and severe cases^9^, is increasing, thus making diagnosis even more difficult. Indeed, according to the Japan AIH National Survey, the prevalence rate per 100,000 people in 2016 was 23.9, which is three times higher than that in 2004 (8.7 per 100,000 people)^10^. Similarly, the prevalence is also increasing in Sweden and the Netherlands^11,12^.

Growth differentiation factor 15 (GDF15), also known as macrophage inhibitory cytokine-1, is one of the growth differentiation promoting factors belonging to the transforming growth factor-beta superfamily and is a cytokine expressed in systemic tissues, and it is considered to regulate cell proliferation^13^. Furthermore, GDF15 increases in life-threatening situations, such as inflammation and infectious diseases^14^; however, the detailed underlying mechanism remains unknown. Notably, GDF15 is useful as a biomarker for mitochondrial disease^15^, and it is elevated in diseases, such as cancer^16^, heart disease^17^, and diabetes^18^. In addition, the relationship between GDF15 and liver disease has been reported in hepatitis C virus (HCV) infection, cirrhosis, hepatocellular carcinoma (HCC)^19^, non-alcoholic fatty liver disease (NAFLD)^20^, and primary biliary cirrhosis (PBC)^21^, and GDF15 is associated with hepatic fibrosis and severity. However, the role of GDF15 in AIH remains unknown. Delayed diagnosis and therapeutic intervention exacerbate cirrhosis and liver failure in AIH patients; therefore, useful diagnostic markers are urgently needed to facilitate early and easy diagnosis. In this study, we aimed to investigate whether GDF15 is useful as a diagnostic and therapeutic marker for AIH; to our knowledge, this is the first such report.

## Materials and methods

### Participants

We enrolled 45 Japanese patients with AIH, 17 patients with hepatitis B (HB), 15 patients with hepatitis C (HC), 20 patients with PBC, and 74 healthy controls at the Kurume University Hospital (Kurume, Japan) from January 2008 to December 2018. All patients were enrolled at the time of diagnosis and were not treated. Patients with HCC, other cancers, heart disease, diabetes, or infections from other than the hepatitis virus were excluded from this study to eliminate other factors resulting in high GDF15 levels.

The study protocol conformed to the ethical guidelines of the 1975 Declaration of Helsinki, and comprehensive informed consent regarding the use of data was obtained from each participant. The study protocol was approved by the institutional review board of Kurume University (Approval No.: 19049).

### Diagnosis and definition

AIH is characterized by elevated transaminase, elevated immunoglobulin G levels, presence of antinuclear antibodies (ANA) or anti-smooth muscle antibody in serum, histological features of interface hepatitis with infiltration of lymphocytes and plasma cells, when other liver diseases (viral hepatitis, drug-induced liver injury, Wilson's disease, hereditary hemochromatosis, etc.) are excluded. Therefore, we diagnosed AIH based on the revised criteria and/or simplified criteria of the IAIHG^22^. All patients with AIH had ANA, and negativity for antibodies to liver-kidney microsomal type-1 antibody was observed. Therefore, the patients were diagnosed with AIH type 1. The diagnosis of PBC was made according to the diagnostic criteria described in the clinical practice guidelines for PBC in Japan. Patients with HB or HC were diagnosed with positive hepatitis B virus deoxyribonucleic acid or HCV ribonucleic acid for at least 6 months, respectively. In the absence of histological analysis, liver cirrhosis was diagnosed based on the presence of thrombocytopenia (< 10 × 10^4^/μL), a low level of serum albumin (< 3.5 g/dL), esophageal–gastric varices, splenomegaly, and compatible imaging features on ultrasonography or computed tomography. Of the 97 patients, 26 (26.8%) were diagnosed with compensated cirrhosis.

### Clinical and laboratory assessments

The following serum parameters were examined: total bilirubin (TB), aspartate aminotransferase (AST), alanine aminotransferase (ALT), lactate dehydrogenase (LDH), gamma-glutamyl transpeptidase (GGT), albumin, platelet counts, prothrombin time-international normalized ratio (PT-INR), Mac-2 binding protein glycan isomer (M2BPGi), type IV collagen, Fib 4 index, immunoglobulin (Ig) M, IgG, alpha-fetoprotein (AFP), ANA, and anti-mitochondrial antibody (AMA). In addition, sex and age were also analyzed.

### Histological assessment

A liver biopsy was performed before the initial treatment. Histological evaluation was performed by two or three pathologists at the Department of Diagnostic Pathology, Kurume University Hospital. Staging and grading were performed according to the classification of Desmet et al.^23^.

### Procedures for GDF15 study

#### Enzyme-linked immunosorbent assay (ELISA)

Serum samples used in this study were collected for analysis from 2008 to 2018. All samples were stored at − 80 °C until analysis. We measured GDF-15 concentrations in duplicate samples using ELISA (R & D Systems, Minneapolis, USA). The samples in which the concentration of GDF-15 was higher than the range of detection were further diluted tenfold with dilution buffer and reanalyzed. The values of each assay were compared with the reference GDF-15 concentrations. All assays were performed by a trained scientist.

#### Immunohistochemistry

Paraffin-embedded liver tissue Sects. (5 μm-thick) were boiled for 30 min in high pH target retrieval solution for antigen retrieval, and subsequently incubated with primary antibodies, and thereafter with secondary antibodies. These antibodies are presented in Supplementary Table 1. Nuclei were stained with DAPI (#H-1200, Vector Laboratories, Inc., CA, USA) for counterstaining. Immunoreactivity was visualized using EnVision + system HRP-labeled polymer anti-rabbit (#K4003, DAKO Japan, Kyoto, Japan) and a DAB commercial kit (Liquid DAB + Substrate Chromogen System, #K3468, DAKO Japan, Kyoto, Japan).

#### Immunohistochemistry scoring

Slides of immunostained tissue sections were evaluated by light microscopy, and the immunohistochemistry signal was scored using the so-called ‘Allred Score’ (Allred et al., 1998). Briefly, a proportion score was assigned representing the estimated proportion of positive staining cells (0 = none; 1 < 1%; 2 = 1% to 10%; 3 = 10% to < 1/3; 4 = 1/3 to < 2/3; 5 =  > 2/3). Average estimated intensity of staining in positive cells was assigned an intensity score (0 = none; 1 = weak; 2 = intermediate; 3 = strong). Proportion and intensity scores were added to obtain a total score that ranged from 0–8.

### Statistical analyses

Data are presented as mean ± standard deviation. Group comparisons were conducted using the t-test, Mann–Whitney U test, or Wilcoxon/Kruskal–Wallis test for continuous variables and the chi-squared test for categorical variables. Spearman’s correlation analysis was used to assess the correlation between GDF15 levels and biochemical data. The area under the receiver-operating-characteristic (ROC) curve was calculated to evaluate the ability to distinguish AIH and other groups. The optimal cutoff was determined by the Youden’s index method.

Data analyses were performed using JMP software version 11.0.0 (SAS, Cary, NC, USA). Statistical significance was set at P < 0.05.

### Compliance with ethical requirements

All authors consented to participate in this series of studies and to publish it. All authors approved the version to be published. All authors agree to be accountable for all aspects of the work in ensuring that questions related to the accuracy or integrity of any part of the work are appropriately investigated and resolved.

### Informed consent in studies with human subjects

All procedures followed were in accordance with the ethical standards of the responsible committee on human experimentation (institutional and national) and with the Helsinki Declaration of 1975, as revised in 2008. Informed consent was obtained from all patients for being included in the study.

### Patent

Y.K. has a patent as “GDF15: a new diagnostic biomarker for mitochondrial diseases” which has been registered as application No. PCT/JP2015/050833 on Jan 14, 2015 and was certificated for patent # 6711966 by Japan Patent Office on June 2, 2020 issued. Patent rights are assigned to the Tokyo Metropolitan Institute of Gerontology and Kurume University. Licensee is MBL.

## Results

### Clinical characteristics of patients with liver diseases

Table 1 shows the characteristics of patients at the time of enrollment in this study. Patients with liver diseases were older than controls (*p* < 0.0001), and patients with AIH were older than those with HB or PBC. The proportion of women with AIH or PBC was higher. Cirrhosis was present in 60% of HC patients, 47% of HB patients, and 32% of AIH patients. However, patients with PBC had no cirrhosis. Patients with AIH had higher TB, AST, ALT, LDH, and IgG levels and lower albumin levels than those with other liver diseases. AIH patients had higher PT-INR than HC and PBC patients and higher M2BPGi and Fib4 index than HB and PBC patients. There were only two patients with primary sclerosing cholangitis; their data are shown in Supplementary Table 2 and were excluded from the statistical analysis.

**Table 1:** Clinical characteristics of patients with liver diseases.

	Healthy control	HB	HC	PBC	AIH
number	74	17	15	20	45
age (years)	35.0 ± 8.3	50.6 ± 11.4	56.1 ± 17.4	53.7 ± 10.6	60.2 ± 12.9
gender(Female:Male)	44:30	6:11	8:7	18:2	39:6
non-LC:LC		9:8	6:9	20:0	31:14
TB (mg/dL)		1.0 ± 0.4	1.0 ± 0.4	0.8 ± 0.3	2.6 ± 3.1
AST (IU)		51.6 ± 19.6	49.3 ± 27.7	42.3 ± 21.8	291.8 ± 448.3
ALT (IU)		61.4 ± 30.6	49.1 ± 35.1	59.6 ± 77.9	364.5 ± 511.0
LDH (U/L)		182.6 ± 32.6	206.0 ± 54.1	192.9 ± 30.8	286.3 ± 155.4
GGT (U/L)		79.4 ± 70.3	62.3 ± 74.0	210.2 ± 172.8	213.3 ± 218.4
Albumin (g/dL)		4.1 ± 0.4	3.9 ± 0.4	4.1 ± 0.3	3.5 ± 0.5
Platelet (× 10^4^/μL)		14.4 ± 6.1	12.8 ± 6.9	23.4 ± 6.3	17.8 ± 6.9
PT(INR)		1.09 ± 0.10	1.04 ± 0.08	0.96 ± 0.06	1.13 ± 0.16
M2BPGi		2.0 ± 2.3	4.9 ± 4.2	0.9 ± 0.4	5.9 ± 4.1
Type IV collagen (ng/mL)		133.0 ± 12.1	185.8 ± 67.5	128.0 ± 52.9	275.9 ± 191.4
Fib4 index		3.3 ± 2.6	4.3 ± 2.6	1.5 ± 0.7	5.9 ± 4.5
IgG (mg/dL)		1592.9 ± 404.3	1917.6 ± 651.9	1575.3 ± 428.7	2667.2 ± 901.6
IgM (mg/dL)		127.4 ± 38.9	134.3 ± 65.1	272.8 ± 178.1	234.2 ± 218.9
AFP (ng/mL)		12.7 ± 19.5	11.3 ± 12.9	3.8 ± 2.8	30.9 ± 66.7
GDF15 (pg/dL)	475.3 ± 145.3	953.2 ± 871.4	1568.0 ± 822.3	643.9 ± 247.0	1994.3 ± 1258.0

HB, hepatitis B; HC, hepatitis C; PBC, primary biliary chorangitis; AIH, autoimmune hepatitis; LC, liver cirrhosis; TB, total bilirubin; AST, asparatate aminotransferase; ALT, alanine aminotransferase; LDH, lactate dehydrogenase; GGT, γ-glutamyl transpeptidase; PT, prothrombin time; INR, international normalized ratio; M2BPGi, Mac-2 binding protein glycosylation isomer; Ig, immunoglobulin; AFP, arfa-fetoprotein; GDF15, growth differentiated factor 15.

### Comparison of serum GDF15 levels in patients with liver diseases

**Figure 1:**
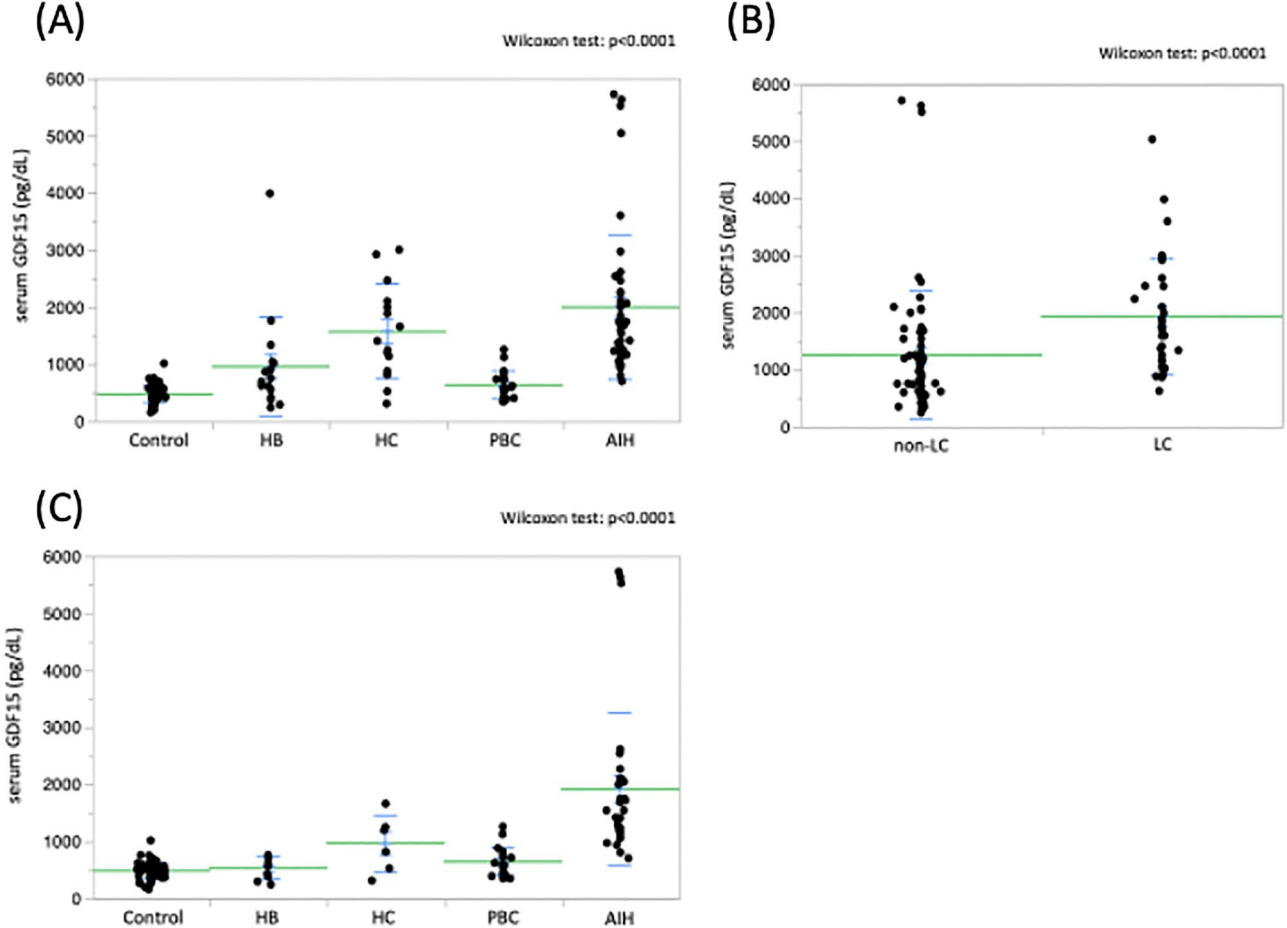
(**A**) Comparison of serum GDF15 levels in patients with liver diseases. Serum GDF15 levels were significantly higher in patients with AIH than in those with HB or PBC and healthy controls (*p* < 0.0001). In addition, serum GDF15 levels were significantly higher in HC patients than in PBC patients and healthy controls (*p* < 0.005). (Wilcoxon test; *p* < 0.0001) (**B**) Comparison of serum GDF15 levels in patients with non-LC and LC. Serum GDF15 levels were significantly higher in patients with LC than in those with non-LC (Wilcoxon test; *p* < 0.0001). (**C**) Comparison of serum GDF15 levels among various liver diseases in patients with non-LC. Serum GDF15 levels were significantly higher in patients with AIH than in those with other liver diseases and healthy controls (*p* < 0.0001 or *p* < 0.005). (Wilcoxon test; *p* < 0.0001). GDF15, growth differentiation factor 15; AIH, autoimmune hepatitis; HB, hepatitis B; HC, hepatitis C; PBC, primary biliary cholangitis; LC, liver cirrhosis.

Serum GDF15 levels in patients with AIH (1994.3 ± 1258.0 pg/dL) were higher than those in patients with HB (953.2 ± 871.4 pg/dL) or PBC (643.9 ± 247.0 pg/dL) and healthy controls (475.3 ± 145.3 pg/dL) (*p* < 0.0001) (Fig. 1A). In addition, serum GDF15 levels were also significantly higher in patients with cirrhosis (1926.6 ± 1026.0 pg/dL) than in those without cirrhosis (1249.1 ± 1124.1 pg/dL) (*p* < 0.0001) (Fig. 1B).

Moreover, as shown in Table 2, serum GDF15 levels were higher in patients with AIH (1914.0 ± 1327.2 pg/dL) than in those with other liver diseases and healthy controls (*p* < 0.0001) (Fig. 1C).

**Table 2:** Clinical characteristics of various liver diseases in patients without cirrhosis.

	Healthy control	HB	HC	PBC	AIH
number	74	9	6	20	31
age (years)	35.0 ± 8.3	44.4 ± 11.6	43.7 ± 17.0	53.7 ± 10.6	57.1 ± 12.2
gender(Female:Male)	40:34	5:4	4:2	18:2	27:4
TB (mg/dL)		0.8 ± 0.2	0.7 ± 0.2	0.8 ± 0.3	3.0 ± 3.6
AST (IU)		42.2 ± 12.4	43.2 ± 13.5	42.3 ± 21.8	350.5 ± 523.3
ALT (IU)		60.4 ± 32.0	49.8 ± 16.7	59.6 ± 77.9	459.3 ± 578.4
LDH (U/L)		167.3 ± 24.4	170.0 ± 32.4	192.9 ± 30.8	296.2 ± 185.6
GGT (U/L)		52.4 ± 73.1	56.7 ± 49.1	210.2 ± 172.8	243.5 ± 248.7
Albumin (g/dL)		4.3 ± 0.3	4.2 ± 0.4	4.1 ± 0.3	3.6 ± 0.4
Platelet (× 10^4^/μL)		18.4 ± 4.3	19.7 ± 5.8	23.4 ± 6.3	19.8 ± 7.0
PT(INR)		1.01 ± 0.05	0.99 ± 0.05	0.96 ± 0.06	1.14 ± 0.17
M2BPGi		1.08 ± 0.77	1.92 ± 1.82	0.94 ± 0.37	5.88 ± 4.37
Type IV collagen (ng/mL)		133.0 ± 12.1	167.0 ± 72.2	128.0 ± 52.9	264.7 ± 207.4
Fib4 index		1.4 ± 0.6	1.7 ± 1.3	1.5 ± 0.7	5.3 ± 4.9
IgG (mg/dL)		1597.3 ± 348.4	1861.8 ± 558.0	1575.3 ± 428.7	2639.1 ± 998.0
IgM (mg/dL)		128.3 ± 38.8	134.8 ± 71.5	272.8 ± 178.1	235.2 ± 240.0
AFP (ng/mL)		3.7 ± 0.6	10.9 ± 10.5	3.8 ± 2.8	39.3 ± 77.3
GDF15 (pg/dL)	475.3 ± 145.3	519.3 ± 197.5	955.7 ± 502.7	634.9 ± 247.0	1914.0 ± 1327.2

HB, hepatitis B; HC, hepatitis C; PBC, primary biliary chorangitis; AIH, autoimmune hepatitis; TB, total bilirubin; AST, asparatate aminotransferase; ALT, alanine aminotransferase; LDH, lactate dehydrogenase; GGT, γ-glutamyl transpeptidase; PT, prothrombin time; INR, international normalized ratio; M2BPGi, Mac-2 binding protein glycosylation isomer; Ig, immunoglobulin; AFP, arfa-fetoprotein; GDF 15, growth differentiated factor 15.

### Correlation of serum GDF15 levels with clinical data in patients with liver diseases

**Figure 2:**
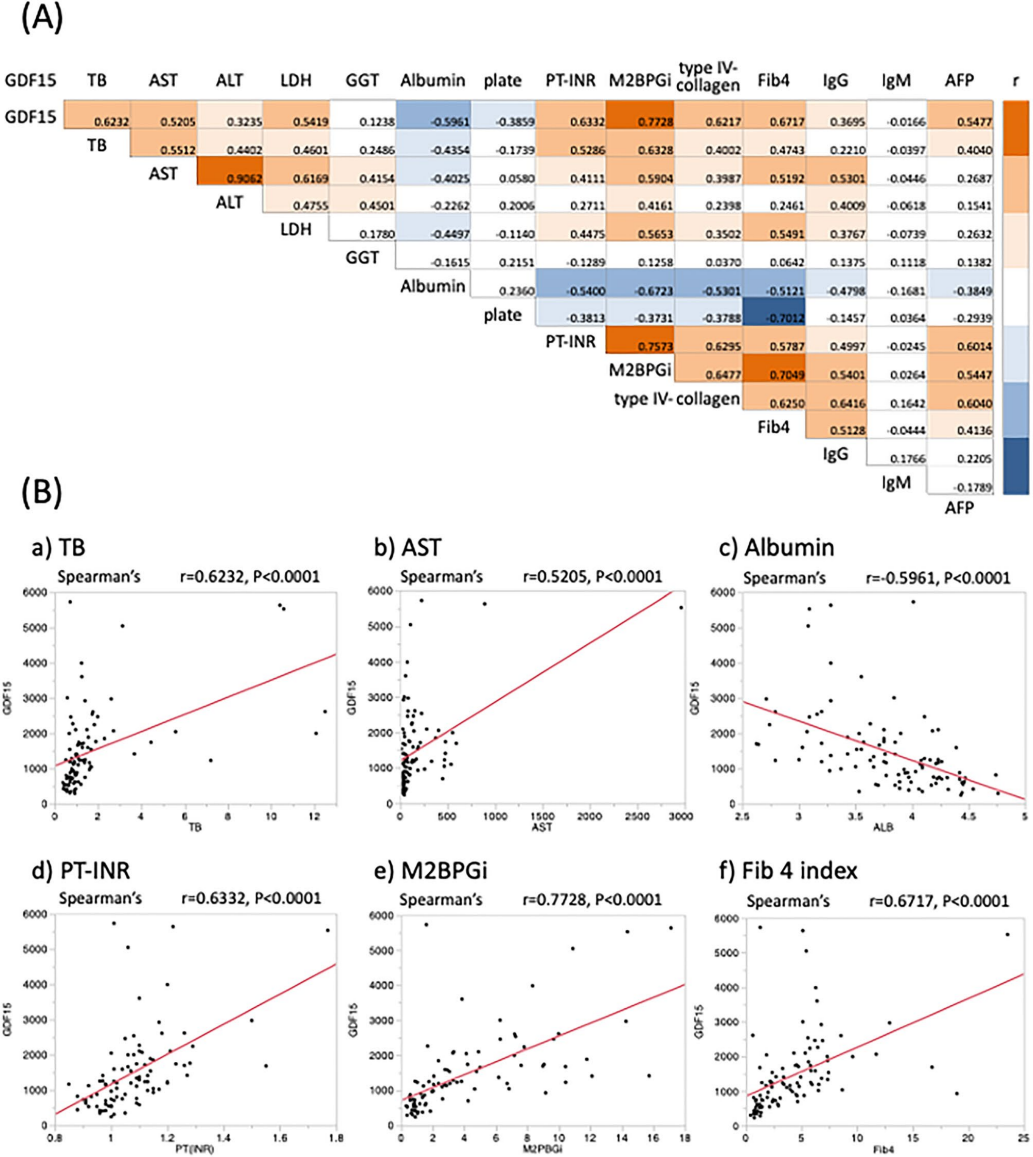
(**A**) Correlation map between the clinical data in patients with liver disease by Spearman’s correlation analysis. The positive correlation between the data is shown in orange, while the negative correlation between the data is shown in blue. (**B**) Correlation of serum GDF15 levels and clinical data in patients with liver disease by Spearman’s correlation analysis. Serum GDF15 levels had significant positive correlations with TB, AST, PT-INR, M2BPGi, and Fib4 index. However, serum GDF15 levels were negatively correlated with albumin levels. GDF15, growth differentiation factor 15; TB, total bilirubin; AST, aspartate aminotransferase; ALT, alanine aminotransferase; PT-INR, prothrombin time-international normalized ratio; M2BPGi, Mac-2 binding protein glycosylation isomer; Ig, immunoglobulin; AFP, alpha-fetoprotein.

Figure 2A shows a correlation map between clinical data of patients with various liver diseases by Spearman's correlation analysis. M2BPGi showed the strongest positive correlation with serum GDF15 levels (r = 0.7728, *p* < 0.0001). In addition, serum GDF15 levels also had a strong positive correlation with TB (r = 0.6232, *p* < 0.0001), AST (r = 0.5205, *p* < 0.0001), PT-INR (r = 0.6332, *p* < 0.0001), and the Fib 4 index (r = 0.6717, *p* < 0.0001). However, serum GDF15 levels were negatively correlated with albumin levels (r = −0.5961, *p* < 0.0001) (Fig. 2B).

### Receiver-operating-characteristic (ROC) curve analysis

**Figure 3:**
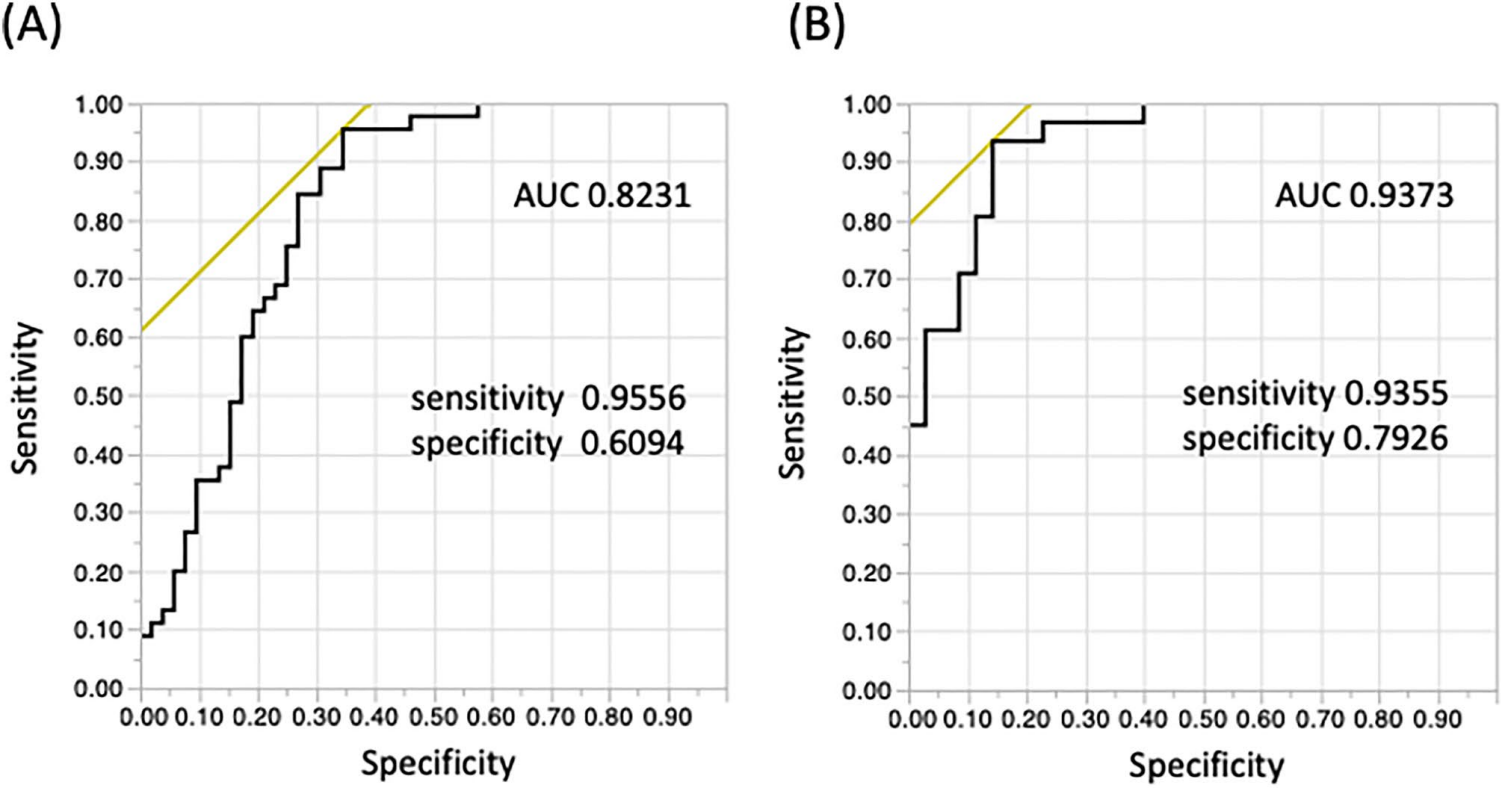
(**A**) ROC curve analysis showed that serum GDF15 levels were useful in distinguishing AIH from other liver diseases, especially non-LC (**B**). (**A**) The AUC was 0.8231, with a sensitivity of 95.6%, specificity of 60.9%, and an optimal cut-off value of 931.0 pg/mL. (**B**) The AUC was 0.9373, with sensitivity of 93.6%, specificity of 79.3%, and an optimal cut-off value of 931.3 pg/mL. ROC, receiver operating characteristic; GDF15, growth differentiation factor 15; AIH, autoimmune hepatitis; LC, liver cirrhosis; AUC, area under the curve.

The area under the ROC curve (AUC) of distinguishing AIH from other liver diseases was 0.8231, with an optimal cutoff value of 931.0 pg/mL, a sensitivity of 95.6%, and a specificity of 60.9% (Fig. 3A). Similarly, the AUC of distinguishing AIH from other liver diseases in non-cirrhosis patients was 0.9373, with an optimal cutoff value of 931.3 pg/mL, a sensitivity of 93.6%, and a specificity of 79.3% (Fig. 3B).

### Comparison of serum GDF15 levels before and after treatment in AIH patients

All 45 AIH patients received prednisolone treatment (0.5–1.0 mg/kg/day), and all had normalized ALT and IgG levels. Serum GDF15 levels were also measured in eight of the 45 patients with AIH after treatment. The mean serum GDF15 levels were significantly lower after treatment than before treatment (mean values; 2174.8 vs. 901.8, *p* = 0.0296).

### Evaluation of GDF15 immunostaining in liver tissues of AIH patients before and after treatment (Fig. 4)

**Figure 4:**
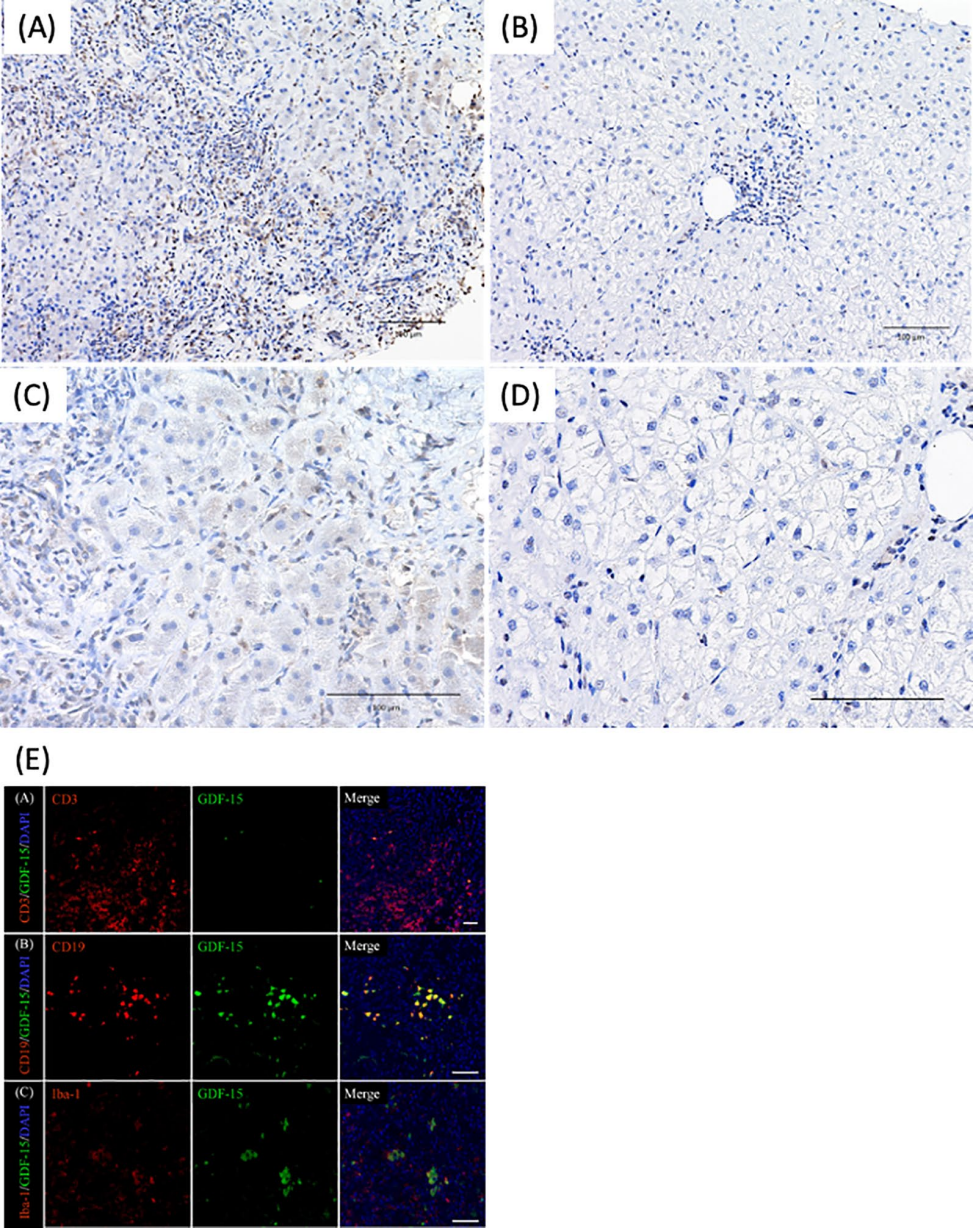
Evaluation of GDF15 immunostaining in liver tissues of AIH patients before (**A**,**C**) and after (**B**,**D**) treatment (**A**,**C**) GDF15 immunostaining revealed hepatic cytoplasm, sinusoidal endothelial cells, and inflammatory cells infiltrating the portal vein area were strongly stained. The immunohistochemistry total score is equivalent to 7. (**B**,**D**) GDF15 staining of liver tissues showed fewer positive cells and weaker staining in inflammatory cells infiltrating the portal vein area after treatment than before treatment. The immunohistochemistry total score is equivalent to 2. (**E**) Double immunofluorescence staining for GDF15 and CD3, CD19, or Iba-1 in the portal area of liver tissues in AIH patient Upper row, immunofluorescence images of GDF15 (green) and CD3 (red). Middle row, immunofluorescence images of GDF15 (green) and CD19 (red). Lower row, immunofluorescence images of GDF15 (green) and Iba-1 (red). The merged images show that GDF15-positive cells are also CD19-positive cells. GDF15, growth differentiation factor 15; AIH, autoimmune hepatitis.

We evaluated GDF15 staining of liver tissues before and after treatment in three AIH patients who had biological remission. GDF15 was strongly stained in liver tissues of patients with AIH before treatment; particularly, hepatic cytoplasm, sinusoidal endothelial cells, and inflammatory cells infiltrating the portal region and the lobule were strongly stained (Fig. 4A,C). However, there were fewer GDF15-positive cells and weaker GDF15 staining after treatment than before treatment (Fig. 4B,D). The immunohistochemical score of GDF15 was 6.3 ± 0.7 before treatment and 2.2 ± 0.4 after treatment, which was a significant decrease (Wilcoxon test; *p* = 0.0002). Furthermore, we performed double immunofluorescence staining to evaluate which types of infiltrating cells are GDF-15-positive in the pathology of AIH (Fig. 4E). Staining revealed that B-lymphocytes (CD-19-positive cells) were GDF-15-positive.

## Discussion

This study showed that serum GDF15 levels were higher in patients with AIH, especially those with non-cirrhosis, than in those with other liver diseases, and the ROC analysis found that serum GDF15 levels could be a diagnostic marker for AIH. In addition, hepatic immunohistological GDF15 staining of AIH revealed that hepatic cytoplasm, sinusoidal endothelial cells, and infiltrating inflammatory cells were GDF15-positive. Furthermore, treatment remission reduced serum GDF15 levels and decreased GDF15-positive cells in the liver tissue. Our findings suggest that, in patients with AIH, GDF15 is produced at the site of hepatitis, and serum GDF15 levels are elevated.

Serum GDF15 levels have been reported to be high in cancer^16,19^, cardiovascular disease^17^, diabetes^18^, metabolic disease, autoimmune disease^24,25^ and renal disease^26^. Thus, patients with these complications were excluded from the study. Accordingly, it was possible to determine the relationship between liver disease and GDF15 more simply and clearly. Previous studies of liver disease reported that serum GDF15 levels increased with the progression of liver fibrosis in HC^19^ and NAFLD^20^. Consistently, in this study, serum GDF15 levels were significantly higher in patients with cirrhosis than in those without cirrhosis, and serum markers of hepatic fibrosis showed a relatively strong positive correlation with serum GDF15 levels. Importantly, serum GDF15 levels for each liver disease were further compared in patients with non-cirrhosis to eliminate the effects of liver fibrosis. We found that serum GDF15 levels were significantly higher in patients with AIH than in those with other liver diseases. GDF15 is expressed in the liver and upregulated during acute liver injury and liver regeneration^14^. In addition, GDF15, also known as an inflammation-related hormone, is induced by inflammation and is necessary for surviving bacterial and viral infections, and sepsis^27^. The pathogenesis and mechanisms of inflammation in AIH have not yet been clarified. The high serum GDF15 level might be due to more severe liver inflammation and prominent liver regeneration in AIH than in other liver diseases. This speculation is supported by the finding that serum GDF15 levels were positively correlated with TB, AST, and PT-INR, which are indicators of liver damage, and were negatively correlated with albumin levels synthesized from hepatocytes. Moreover, although the number of AIH cases examined was small, the significant decrease in serum GDF15 levels after treatment compared with before treatment and the histopathological findings of GDF15 also support this speculation.

The hepatic pathological findings of AIH have demonstrated that inflammatory cell infiltration is prominent and extends to the lobule, as well as the portal vein area^1,28,29^. The portal vein area is significantly expanded by the infiltration of inflammatory cells and the proliferation of fibers, but the improvement of liver inflammation reduces the infiltration of inflammatory cells and attenuates the expansion of the portal vein area. In AIH, severe hepatic fibrosis has been reported to improve after treatment in more than half of the patients^29,30^. In our study, immunohistological staining of the liver revealed that inflammatory cells infiltrating the portal area were GDF15-positive (the majority of them were B lymphocytes), and hepatic cytoplasm and sinusoidal endothelial cells were also GDF15-positive in AIH patients. In the liver tissue in AIH patients with remission, GDF15-positive cells were markedly reduced. These results suggest that serum GDF15 levels are associated with liver tissue inflammation, degree of tissue damage, and liver regeneration.

This study has some limitations. First, atypical AIH was not examined. Further investigation of atypical AIH, such as acute onset AIH and drug-related AIH, is important and should clarify whether GDF15 can distinguish atypical AIH from acute hepatitis and drug-induced hepatitis. Second, the number of serum and liver GDF15 measurements for AIH patients after treatment was low. Further studies evaluating serum and liver GDF15 measurements for a larger cohort of AIH patients before and after treatment are warranted to determine whether the GDF15 level is useful for assessing remission and predicting prognosis.

In conclusion, this study suggests that GDF 15 is a useful, novel diagnostic and therapeutic biomarker for AIH in chronic liver disease. Further studies are needed to clarify whether GDF15 can predict relapse, refractory, and poor prognosis in AIH patients.

## Supplementary Information

Supplementary Information 1.

Supplementary Information 2.

## Data Availability

Upon request, we are prepared to send relevant documents or data (raw data, samples, records, etc.) to verify the validity of the presented results.

## Abbreviations

AIH: Autoimmune hepatitis
IAIHG: International AIH group
GDF15: Growth differentiation factor 15
HCV: Hepatitis C virus
HCC: Hepatocellular carcinoma
NAFLD: Non-alcoholic fatty liver disease
PBC: Primary biliary cholangitis
HB: Hepatitis B
HC: Hepatitis C
ANA: Antinuclear antibodies
TB: Total bilirubin
AST: Aspartate aminotransferase
ALT: Alanine aminotransferase
LDH: Lactate dehydrogenase
GGT: γ-Glutamyl transpeptidase
PT: Prothrombin time
INR: International normalized ratio
M2BPGi: Mac-2 binding protein glycosylation isomer
Ig: Immunoglobulin
AFP: Alpha-fetoprotein
AMA: Anti-mitochondrial antibody
ELISA: Enzyme-linked immunosorbent assay
ROC curve: Receiver operating characteristic curve
AUC: Area under the curve
pAb: Polyclonal antibody
mAb: Monoclonal antibody
LC: Liver cirrhosis
