# Supplementary material for: Growth differentiation factor 15 as a novel diagnostic and therapeutic marker for autoimmune hepatitis
Source: Sci Rep. 2022 May 24;12:8759. doi: 10.1038/s41598-022-12762-9 (PMC9130300; doi:10.1038/s41598-022-12762-9)
Supplement: Supplementary file 2 — Supplementary Information 2. [file 41598_2022_12762_MOESM2_ESM.docx]

Supplementary Table 2. Clinical characteristics of two patients with primary sclerosing cholangitis

| case | 1 | 2 |
| --- | --- | --- |
| age (years) | 24 | 29 |
| gender | Male | Female |
| non-LC or LC | non-LC | non-LC |
| TB (mg/dL) | 0.9 | 1.1 |
| AST (IU) | 64 | 84 |
| ALT (IU) | 32 | 159 |
| LDH (U/L) | 224 | 185 |
| GGT (U/L) | 290 | 729 |
| Albumin (g/dL) | 4.8 | 4.2 |
| Platelet (x10^4^/μL) | 31.7 | 31.2 |
| PT(INR) | 1 | 0.99 |
| M2BPGi | 0.3 | 0.8 |
| Type IV collagen (ng/mL) | 67 | 82 |
| Fib4 index | 0.9 | 0.6 |
| IgG (mg/dL) | 1479 | 2111 |
| IgM (mg/dL) | 149 | 111 |
| AFP (ng/mL) | no test | 3.2 |
| GDF15 (pg/dL) | 519.5 | 489.4 |
|  |  |  |

Abbreviations

LC, liver cirrhosis; TB, total bilirubin; AST, asparatate aminotransferase; ALT, alanine aminotransferase; LDH, lactate dehydrogenase; GGT, γ-glutamyl transpeptidase; PT, prothrombin time; INR, international normalized ratio; M2BPGi, Mac-2 binding protein glycosylation isomer; Ig, immunoglobulin; AFP, arfa-fetoprotein; GDF15, growth differentiated factor 15.
